# ROS-responsive adhesive nanocomposite hydrogel promotes tendon-to-bone healing by modulating the inflammatory microenvironment and pro-differentiation

**DOI:** 10.1093/rb/rbaf107

**Published:** 2025-11-04

**Authors:** Shaowei Zheng, Jiang Guo, Lin Li, Yang-Chi-Dung Lin, Peng Zhang, Wenqiang Li, Xintao Zhang

**Affiliations:** Department of Sports Medicine and Rehabilitation, Peking University Shenzhen Hospital, Shenzhen, Guangdong 518036, P.R. China; Department of Sports Medicine and Rehabilitation, Shenzhen Xinhua Hospital (Shenzhen Sports Hospital), Shenzhen, Guangdong 518038, P.R. China; Department of Sports Medicine and Rehabilitation, Peking University Shenzhen Hospital, Shenzhen, Guangdong 518036, P.R. China; Department of Sports Medicine and Rehabilitation, Peking University Shenzhen Hospital, Shenzhen, Guangdong 518036, P.R. China; Department of Medicine, Shenzhen University, Shenzhen, Guangdong 518060, P.R. China; School of Medicine, The Chinese University of Hong Kong, Shenzhen, Guangdong 518172, P.R. China; Warshel Institute for Computational Biology, School of Medicine, The Chinese University of Hong Kong, Shenzhen, Guangdong 518172, P.R. China; Guangdong Provincial Key Laboratory of Digital Biology and Drug Development, The Chinese University of Hong Kong, Shenzhen, Guangdong 518172, P.R. China; Department of Endocrinology, Key Laboratory of Endocrinology of National Health Commission, Peking Un-ion Medical College Hospital, Chinese Academy of Medical Sciences & Peking Union Medical College, Beijing 100730, P.R. China; Department of Sports Medicine and Rehabilitation, Peking University Shenzhen Hospital, Shenzhen, Guangdong 518036, P.R. China; The Affiliated Guangdong second Provincial General Hospital of Jinan University, Guangzhou, Guangdong 510315, P.R. China; Department of Sports Medicine and Rehabilitation, Peking University Shenzhen Hospital, Shenzhen, Guangdong 518036, P.R. China

**Keywords:** Cu-Zn bimetallic-organic framework, adhesive nanocomposite hydrogel, ROS responsiveness, modulation of inflammatory microenvironment, tendon-to-bone healing

## Abstract

Natural functional regeneration of the tendon–bone interface in rotator cuff repair surgery remains a major challenge and requires the development of innovative therapeutic strategies. Hydrogels with biomechanical adaptability and regenerative microenvironment modulation are promising candidates for treating such injuries. In this study, an ROS-responsive adhesive nanocomposite hydrogel (TPGA@CZB) was developed to enhance tendon-to-bone interface repair by on-demand drug release to modulate the inflammatory microenvironment and promote cell differentiation. The hydrogel consisted of baicalin (Ba)-loaded Cu-Zn bimetallic-organic framework (CZB), N-[tris(hydroxymethyl) methyl] acrylamide (THMA), poly(ethylene glycol) diacrylate (PEGDA) and phenylboronic acid modified methacrylated gelatin (GelMA-CPBA). Owing to its multi-crosslinked structure, TPGA@CZB exhibits excellent adhesive properties (lap shear strength reaching 110.90 ± 15.38 kPa) and mechanical adaptability (compressive strain exceeding 80% and tensile strain of 196.24 ± 3.87%). Additionally, TPGA@CZB demonstrated favorable ROS-responsive release characteristics, with the cumulative release of Ba in H_2_O_2_ solution (63.90 ± 4.76% at 96 h) being significantly higher than that in PBS solution (48.39 ± 1.56% at 96 h). Furthermore, cellular experiments revealed that TPGA@CZB effectively scavenged intracellular ROS, inhibits the NF-κB signaling pathway, regulates macrophage polarization and promotes osteogenic differentiation and chondrogenesis. *In vivo* studies confirmed that TPGA@CZB treatment effectively optimized collagen remodeling, enhanced osteogenesis and cartilage formation, as well as modulated the inflammatory microenvironment at the injury site. In conclusion, this nanocomposite hydrogel integrates “mechanical support—controlled drug release—microenvironment regulation” into a single platform, offering a promising multifunctional therapeutic strategy for enhancing tendon–bone interface regeneration.

## Graphical Abstract

**Figure:**
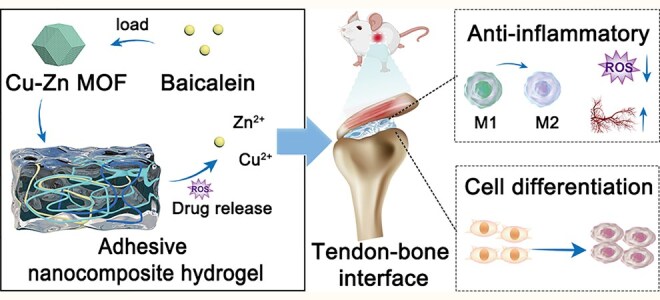

## Introduction

Rotator cuff tears are a leading cause of shoulder pain and disability among middle-aged and elderly individuals, typically resulting in severe pain and impaired mobility [1, 2]. Surgical suturing to alleviate pain and restore shoulder function is the conventional treatment strategy to managing rotator cuff tears [3]. However, the success ratio of current standard surgical repair is remarkably low. Postoperative healing at the tendon–bone interface often forms disorganized fibrovascular scar tissue rather than a natural, functional enthesis structure, leading to a high retear ratio [4–7]. Developing novel tissue engineering strategies to achieve native functional regeneration of the tendon–bone interface remains a significant challenge.

The failure regeneration of fibrocartilage interface is usually associated with the local inflammatory microenvironment, oxidative stress, insufficient blood supply and inadequate recruitment of mesenchymal stem cells [3]. During the early healing phase, the implantation of surgical sutures inevitably triggers severe inflammatory responses. Additionally, the tendon–bone interface is physiologically deficient in angiogenesis, limiting the blood supply and nutrient support available for tissue repair. Inflammatory responses and ischemia lead to excessive reactive oxygen species (ROS) production, which damages cells, impairs tissue repair and further amplifies the inflammatory cascade [1, 3, 4]. Therefore, providing appropriate angiogenesis and establishing an anti-inflammatory microenvironment during the early healing stage are critical for successful tendon–bone regeneration.

Baicalein (Ba), a flavonoid compound extracted from the traditional Chinese herb *Scutellaria baicalensis*, exhibits significant potential in promoting tissue repair owing to its anti-inflammatory, antioxidant and antibacterial effects, coupled with low toxicity [8–10]. For instance, Qin et al. demonstrated that a Ba-based nano-delivery system restored mitochondrial homeostasis via the PPAR signaling pathway, thereby promoting diabetic wound healing [8]. Xiao et al. reported that Ba exerts anti-inflammatory effects in chronic obstructive pulmonary disease by suppressing HIF1A-mediated oxidative stress and apoptosis in bronchial epithelial cells and modulating CD8^+^ T cell cytotoxicity [11]. However, the therapeutic efficacy of Ba is restricted by poor solubility and low bioavailability, highlighting the need for advanced delivery strategies. Zinc-based metal-organic frameworks (ZIF-8), as nanocarriers, have garnered widespread attention in drug delivery due to their high specific surface area, tunable porosity and intelligent controlled-release properties [12–15]. Recent studies have reported that bimetallic metal-organic frameworks (MOFs) demonstrate superior adsorption capacity and higher loading efficiency compared to ZIF-8 alone [16, 17]. Moreover, bioactive metal ions such as Zn^2+^ and Cu^2+^ play crucial regulatory roles in physiological processes such as metabolic regulation and tissue regeneration. For instance, Zn^2+^ exhibits multiple biological benefits for tendon–bone healing, including immunomodulation, pro-angiogenic effects, antibacterial activity, promotion of cell migration and osteogenesis in bone tissue engineering [18, 19]. Similarly, Cu^2+^ demonstrates significant pro-angiogenic and antibacterial properties in biomedical applications [20, 21]. Therefore, employing Cu-Zn MOF (CZ) to deliver Ba represents an ideal strategy to improve its solubility and bioavailability. This system not only enables efficient loading and controlled release of Ba but also degrades to release Zn^2+^ and Cu^2+^, generating synergistic therapeutic benefits for enhanced rotator cuff repair. Specifically, the antioxidant enzyme-like activities of Zn^2+^ and Cu^2+^ may potentiate the free radical scavenging capacity of Ba, establishing a robust defense system against oxidative stress. Concurrently, the multi-target effects of Zn^2+^, Cu^2+^ and Ba on inflammatory signaling pathways can amplify anti-inflammatory efficacy, achieving more profound and sustained anti-inflammatory outcomes.

Considering the unique gradient modulus structure and dynamic mechanical environment of the tendon–bone interface, its long-term repair process requires comprehensive consideration of biomechanical compatibility and regenerative microenvironment regulation. In this regard, hydrogels with high adhesiveness, high elasticity and high flexibility demonstrate significant advantages [22–25]. N-[Tris(hydroxymethyl)methyl] acrylamide (THMA) is a high-performance adhesive material whose monomer contains multiple hydrogen bond donors and acceptors, enabling the formation of extensive hydrogen bonds on and within the hydrogel, significantly enhancing interfacial bonding strength [26, 27]. Meanwhile, the dynamic reversible hydrogen bond network provides an additional energy dissipation mechanism, further improving adhesion performance. However, THMA lacks sufficient mechanical strength and tensile properties as a tendon–bone interface healing material, necessitating the introduction of novel polymer networks to enhance its elasticity and toughness. Additionally, to improve biosafety and prevent excessive angiogenesis that exacerbates scar formation, developing an intelligent drug delivery system capable of precisely controlled release is critical [3, 5, 28, 29]. Boronate ester bonds are dynamically reversible covalent bonds with ROS sensitivity that undergo cleavage upon ROS triggering, enabling the release of encapsulated drugs [30]. Incorporating boronate ester bonds into hydrogels allows for the fabrication of dynamically controlled drug delivery systems, while simultaneously enhancing the mechanical properties of the hydrogels to improve therapeutic efficacy.

**Figure 1:**
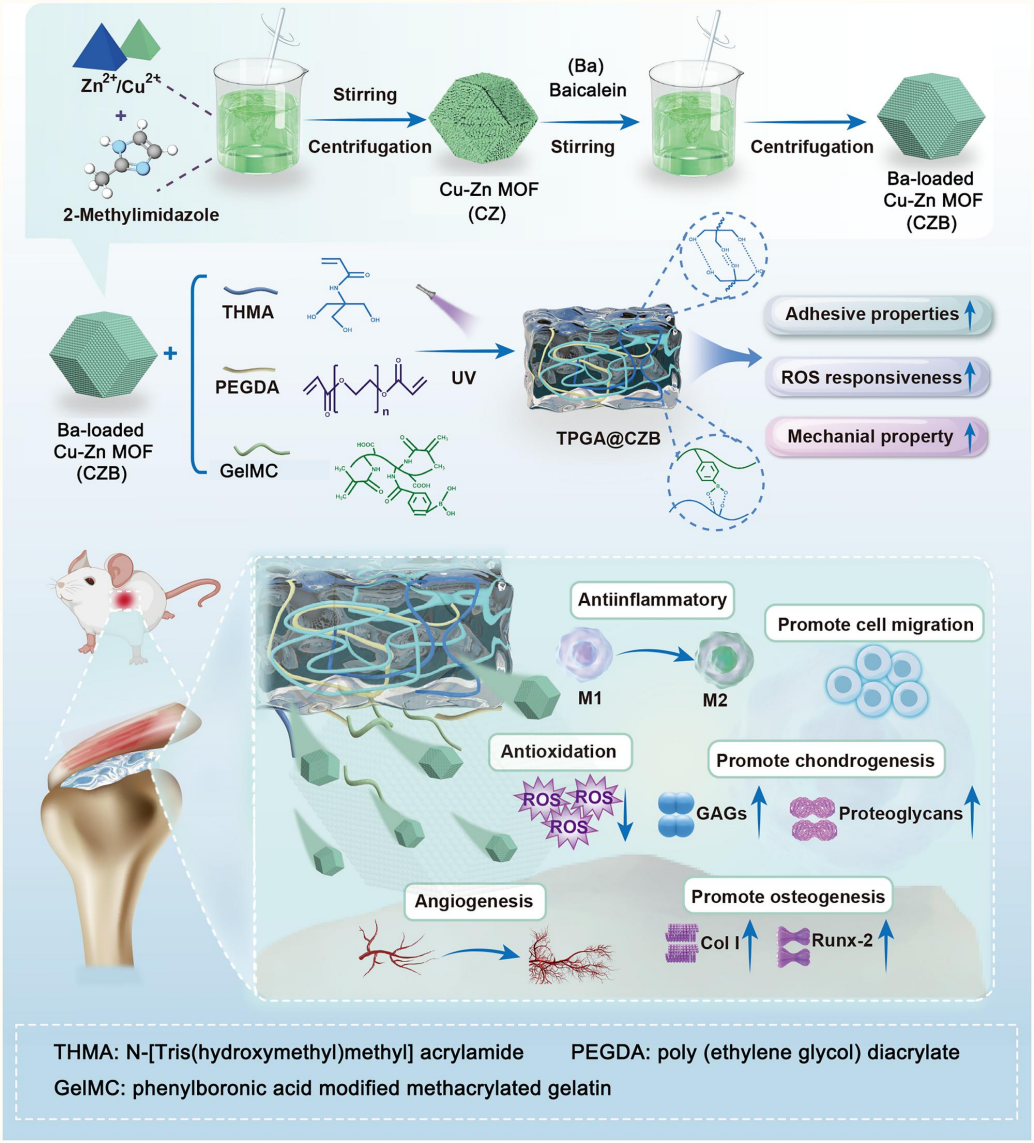
Preparation of TPGA@CZB and its role in tendon–bone healing.

In this study, an ROS-responsive adhesive nanocomposite hydrogel was developed for promoting tendon–bone healing. As illustrated in Figure 1, CZ was first synthesized via a coprecipitation method, followed by electrostatic adsorption of Ba to obtain drug-loaded nanoparticles (CZB). Subsequently, CZB was homogeneously dispersed in a precursor solution consisting of THMA, poly(ethylene glycol) diacrylate (PEGDA) and phenylboronic acid modified methacrylated gelatin (GelMA-CPBA), which was photoinitiated and polymerized to form a three-dimensional cross-linked network hydrogel (TPGA@CZB). The hydrogel contains multiple cross-linking structures such as photopolymerized covalent cross-links, dynamic boronate ester linkages and hydrogen bonds. This hybrid crosslinking system endowed the hydrogel with excellent adhesive properties and mechanical performance, enabling maintained contact with the tendon–bone interface and providing necessary mechanical support during healing. Notably, the ROS-sensitive dynamic boronate ester bonds allowed controlled drug release in response to elevated ROS levels at the healing site. The released Ba, Zn^2+^ and Cu^2+^ could provide a suitable anti-inflammatory microenvironment for tendon–bone interface repair by attenuating oxidative stress, modulating macrophage polarization and angiogenesis, while promoting cell migration, chondrogenic differentiation and osteogenic differentiation. This multifunctional therapeutic strategy integrating “mechanical support—controlled drug release—microenvironment regulation” demonstrates significant potential for clinical applications in tendon–bone interface regeneration.

## Methods

### Materials

Polyethylene Glycol (PEG, M*n* = 6000), THMA, 2-methylimidazole and photoinitiator (I2959) were supplied by Sigma-Aldrich (Shanghai) Trading Co., Ltd (Shanghai, China); gelatin, methacrylic anhydride, 4-carboxyphenylboronic acid and Ba were supplied by Shanghai Macklin Biochemical Co., Ltd (Shanghai, China); Zn (No_3_)_2_·6H_2_O and Cu (No_3_)_2_·3H_2_O were purchased from Shanghai Jieshikai Biotechnology Co., Ltd (Shanghai China).

### Preparation of PEGDA

PEGDA with molecular weight of 6000 was synthesized according to the reported method [26]. Briefly, PEG (M*n* = 6000) was mixed with acryloyl chloride in anhydrous methylene chloride solvent under nitrogen atmosphere to form PEGDA. The successful synthesis of PEGDA was demonstrated through Proton Nuclear Magnetic Resonance (^1^H NMR) along with Fourier Transform Infrared Spectroscopy (FTIR) characterization.

### Preparation of GelMA-CPBA

A solution of gelatin (10 g) in sodium bicarbonate buffer (100 mL) was prepared by stirring at 60°C until complete dissolution. Afterward, 0.375 mL of methacrylic anhydride was added dropwise, followed by pH adjustment to 9.0 with NaOH solution. The solution was stirred for 3 h, neutralized with 1.0 M HCl and then subjected to dialysis and freeze-drying, yielding methacrylated gelatin (GelMA).

Next, GelMA-CPBA was synthesized through EDC/NHS-mediated carboxyl group activation. Briefly, an even solution was created by dissolving 0.5 g of GelMA in 25 mL of deionized water. Separately, 4-carboxyphenylboronic acid (1.0 mmol), EDC (1.0 mmol), NHS (1.0 mmol), DMF (9 mL) and water (6 mL) were mixed and reacted at room temperature for 30 min. After dropwise addition of the activated CPBA solution into the GelMA solution, the pH was adjusted to 6.5 with NaOH. The mixture was then stirred for 24 h, dialyzed, and finally, freeze-dried to obtain GelMA-CPBA. Successful synthesis of GelMA-CPBA was verified by ^1^HNMR.

### Preparation of CZB

Zn (No_3_)_2_·6H_2_O (2.14 g) as well as Cu (No_3_)_2_·3H_2_O (0.3479 g) were sequentially dissolved in a 10 ml mixed solution of DMF and methanol (4:1), and sonicate until completely dissolved. Meanwhile, 2-methylimidazole was dissolved in methanol solution and sonicated until fully dissolved. The mixture of Zn (No_3_)_2_·6H_2_O and Cu (No_3_)_2_·3H_2_O was slowly poured into the 2-methylimidazole solution while being constantly stirred for 1 h. Finally, the precipitate was isolated by centrifugation to obtain Cu-Zn MOF (CZ).

The resulting CZ were dispersed in 30 ml of methanol solution. Under continuous stirring and light protection, 10 ml of Ba-methanol solution (4 mg/mL) was added dropwise, followed by stirring for 6 h. Ba-doped Cu-Zn MOF (CZB) were obtained by centrifuging the precipitate, washing with methanol, and then, drying at 37°C.

The physicochemical properties of CZB nanoparticles were systematically characterized with transmission electron microscopy (TEM), dynamic light scattering (DLS), X-ray diffraction (XRD) and Brunauer–Emmett–Teller (BET) analysis.

Additionally, a certain amount of CZB (*m*_0_) was completely dissolved using a dilute nitric acid solution and then centrifuged. The resulting residue was the loaded Ba. The Ba was freeze-dried and weighed (*m*_B_). The loading ratio (LR) of Ba in CZB was calculated using the following formula:


$$\mathrm{LR (\%)}=\frac{m_{B}}{m_{0}}\times 100\%.$$

### Preparation of TPGA@CZB

THMA (15 wt%), PEGDA (1 wt%), GelMA-CPBA (10 wt%), photoinitiator I2959 (0.2 wt%) as well as CZB nanoparticles (0.4 wt%) were uniformly blended to obtain the hydrogel precursor mixture, and UV illumination was applied for 10 min to obtain the TPGA@CZB. TP and TPGA were prepared using the same method. TP consisted of THMA (15 wt%), PEGDA (1 wt%) and photoinitiator I2959 (0.2 wt%), while TPGA comprised THMA (15 wt%), PEGDA (1 wt%), GelMA-CPBA (10 wt%) and photoinitiator I2959 (0.2 wt%).

### Characterization of TPGA@CZB

The lyophilized TPGA@CZB hydrogels were subjected to liquid nitrogen embrittlement, and their cross-sections were sputter-coated with gold for observation of the hydrogel network structure.

To evaluate the swelling properties of the hydrogels, TP, TPGA and TPGA@CZB hydrogel samples (*n *= 5) with a volume of 500 μl each were prepared. After recording the initial weight, the hydrogels were incubated in PBS at 37°C. Their mass was recorded at set time intervals until no further significant changes were observed (equilibrium). The swelling ratio was determined as follow:


$$\mathrm{Swelling}\;\mathrm{ratio}\;(\%)=\frac{W_{t}-W_{0}}{W_{0}}\times 100\%.$$

*W*_t_ and *W*_0_ represent the weight of the hydrogel at a specific time and its initial weight, respectively.

To evaluate the *in vitro* degradation properties of TPGA@CZB, 1 mL of the hydrogel was immersed in PBS solution (pH 7.4) containing 1 mg/mL lysozyme. At predetermined time points, the hydrogels were retrieved, freeze-dried and weighed (*n *= 5). The degradation ratio of TPGA@CZB was calculated using the following formula:


$$\mathrm{Degradation}\;\mathrm{ratio (\%)}=\frac{W_{0}-W_{t}}{W_{0}}\times 100\%.$$

*W_0_* denotes the initial weight of TPGA@CZB, while *W_t_* denotes the weight of TPGA@CZB at a specific point in time.

To evaluate the rheological properties of the hydrogels, a time sweep test was performed on the hydrogel precursor solution using a rheometer equipped with a UV cross-linking device under 10 mW/cm^2^ UV irradiation, with parameters set at 1.0% strain, 10.0 rad/s angular frequency and 37°C.

The hydrogels’ mechanical properties were assessed through compression test and tensile test. For compression testing, cylindrical hydrogel samples (10 mm diameter × 8 mm height) were compressed at 2 mm/min. For tensile testing, rectangular hydrogel specimens (10 mm length × 5 mm width × 3 mm thickness) were stretched at 10 mm/min.

To evaluate the flexibility and adhesion of TPGA@CZB. TPGA@CZB precursor solution (200 μL) was dropped on pig skin and finger joints, respectively. After UV irradiation for 10 min, the TPGA@CZB was observed under various conditions, including bending, stretching and twisting distorting of the pigskin, as well as finger extension and flexion. In addition, the adhesion of the hydrogel samples to the different substrates, such as butadiene-acrylonitrile copolymer, plastics, pig skin, lumber, iron, glass and rubber, was observed. Furthermore, to assess the bonding strength of TPGA@CZB, the hydrogel was placed between a glass slide and a rectangular piece of pigskin. A weight was applied to the overlapping area for 5 min followed by a lap-shear test.

To investigate the ROS-responsive release behavior of TPGA@CZB, 2 mL of TPGA@CZB was sealed in a dialysis bag and placed in PBS (20 mL, pH 7.4) either with or without 1 mM H_2_O_2_, with continuous stirring on a magnetic stirrer. At predetermined time points, PBS samples were collected and the released Ba was quantified by measuring absorbance at 380 nm. Concurrently, the release of Cu^2+^ and Zn^2+^ in the solution was detected via inductively coupled plasma mass spectrometry (ICP-MS). The release profile of Ba was fitted with mathematical models to further explore the release mechanism of Ba.

### Antioxidant test

The hydrogel’s antioxidant capacity was examined via DPPH and ABTS assays. First, 1 mL of TP, TPGA and TPGA@CZB hydrogels were prepared into hydrogel extracts according to the reagent kit instructions. In the DPPH assay, each hydrogel extract was mixed with the DPPH working solution and kept in the dark for 30 min. Afterward, the reaction mixture was subjected to wavelength scanning (400–800 nm), and DPPH clearance ratio was determined from the absorbance change at 517 nm.

In the ABTS assay, the hydrogel extracts were separately reacted with the ABTS^+^**·** working solution and protected from light for 10 min. Absorbance was scanned from 400 to 800 nm, and ABTS^+^**·**radical scavenging activity was determined by monitoring the absorbance variation at 734 nm.

### Antimicrobial test

A 500 μL volume of TP, TPGA or TPGA@CZB hydrogel samples was co-cultured with 2 mL of *Staphylococcus aureus* (*S. aureus*) or *Escherichia coli* (*E. coli*) bacterial suspension (1 × 10^6^ CFU/mL), respectively. After 8 h of co-culture, bacterial suspension samples were collected, diluted and spread onto LB agar plates. Following 24 h of incubation, bacterial colony growth on the solid medium was observed.

### *In vitro* biocompatibility assessment

To evaluate cytocompatibility, hydrogel extracts of TP, TPGA or TPGA@CZB were prepared in accordance with ISO 10993-5 and previous studies by immersing the materials at a concentration of 0.1 g/ml in serum-free DMEM medium, followed by incubation at 37°C for 48 h [31–33]. The resulting extracts were sterilized using a 0.2 μm filter and stored at 4°C until further use. Mouse pre-osteoblast cell line (MC3T3-E1, purchased from SUNNCELL, Wuhan, China) were plated in 96-well plates and maintained until 80% fusion was achieved. The cells were then treated with TP, TPGA or TPGA@CZB hydrogel extracts. At the same time, cells cultured under normal conditions were set as the control group. Cell viability was evaluated after 24 and 48 h of treatment using CCK-8 as well as live/dead cell staining assays. Additionally, at 48 h of culture, phalloidin staining was performed to observe the cytoskeleton.

The hemocompatibility of hydrogels was assessed via hemolysis assay. Specifically, 1.5 mL of fresh whole blood was collected from mice and diluted with 3.5 mL of saline. The mixture was centrifuged and washed, with this process repeated five times to obtain purified red blood cells (RBCs). The RBC suspension (100 μL) was then treated for 2 h at 37°C with saline (negative control), deionized water (positive control), TP extract, TPGA extract or TPGA@CZB extract (900 μL). After centrifugation, the supernatant’s absorbance was measured at 540 nm.

### Intracellular ROS scavenging

To evaluate the ROS-scavenging ability of hydrogels, MC3T3-E1 cells were exposed to oxidative stress by treatment with 200 μM H_2_O_2_. In brief, the cells were plated in 24-well plates and cultured until reaching optimal confluence prior to H_2_O_2_ treatment. After 2 h of treatment, the medium containing TP, TPGA or TPGA@CZB hydrogel extract was replaced. Following 6 h of additional incubation, intracellular ROS were labeled with DCFH-DA (10 μM) and examined by fluorescence microscopy.

### *In vitro* immunomodulatory effect

The role of TPGA@CZB in modulating the inflammatory microenvironment *in vitro* was investigated using murine monocyte-macrophage leukemia cells (RAW264.7, purchased from SUNNCELL, Wuhan, China). Briefly, RAW264.7 cells were first polarized to the M1 phenotype by pretreatment with LPS (10 μg/mL) for 24 h, followed by treatment with fresh medium containing TP, TPGA or TPGA@CZB hydrogel extracts. After 48 h of treatment, the polarization status of macrophages was evaluated by detecting the expression levels of M1 (CD86) and M2 (CD206) markers in RAW264.7 cells via immunofluorescence staining. The culture supernatant from each group was collected, and the levels of inflammatory cytokines (IL-6, TNF-α and IL-10) in the medium were measured by Elisa assays. Additionally, nuclear translocation of p65 in differently treated RAW264.7 cells was examined through immunofluorescence staining to assess the effect of the hydrogel on the NF-κB signaling pathway in RAW264.7 cells.

### The role of TPGA@CZB in promoting angiogenesis *in vitro*

The effect of TPGA@CZB on human umbilical vein endothelial cells (HUVECs, purchased from SUNNCELL, Wuhan, China) migration was investigated using a scratch assay. HUVECs were cultured in 24-well plates until reaching approximately 90% confluency, followed by treatment with 200 μM H_2_O_2_ for 2 h. Subsequently, a scratch was made in the cell monolayer. After washing three times with PBS, the scratched areas were photographed. The cells were then incubated for an additional 24 and 48 h in medium containing extracts of TP, TPGA or TPGA@CZB and cell migration was observed.

The effect of TPGA@CZB on tube formation of HUVECs was investigated by tube formation assay. After being treated with 200 μM H_2_O_2_ for 2 h, HUVECs were seeded into Matrigel-coated 24-well plates and cultured in medium containing TP, TPGA or TPGA@CZB extract. The formation of cellular tubular structures was observed after 24 h of incubation.

The expression level of vascular endothelial growth factor (VEGF) in HUVECs was detected by immunofluorescence staining. After HUVECs were treated with 200 μM H_2_O_2_ for 2 h, the culture medium containing TP, TPGA or TPGA@CZB extracts was added. Following 24 h of incubation, the cells were fixed and incubated with anti-VEGF primary antibody overnight at 4°C. Subsequently, the secondary antibody was applied, and the nuclei were stained with DAPI. VEGF expression was then observed using a fluorescence microscope.

### The role of TPGA@CZB in promoting osteogenesis *in vitro*

MC3T3-E1 cells were seeded into 12-well plates. After cell attachment, cells were treated with 2 μM H_2_O_2_ for 2 h to simulate an oxidative stress environment. Subsequently, the medium was replaced with medium containing TP, TPGA or TPGA@CZB hydrogel extracts. Medium was changed every two days for a total of 21 days. Cells were harvested on Day 7 and 21 post-treatment. Total RNA was extracted using an RNA extraction kit and reverse transcribed into cDNA. Relative expression levels of the early osteogenic marker Runx2 were detected by real-time quantitative PCR on Day 7, while expression levels of late osteogenic markers OCN were assessed on Day 21. Additionally, after 21 days of treatment, cells were fixed and stained with Alizarin red S (ARS) solution for 20 min. Calcium nodule formation was subsequently examined under a fluorescence microscope.

Furthermore, the effect of TPGA@CZB on MC3T3-E1 cells migration was assessed by transwell assay. In brief, MC3T3-E1 cells were cultured to approximately 80% confluence and serum-starved for 12 h. Subsequently, the medium was replaced with serum-free medium containing 200 μM H_2_O_2_ for 2 h to induce oxidative stress. After trypsinization, cell suspensions were prepared in serum-free medium (1 × 10^5^ cells/mL) and transferred to the upper transwell compartment, with TP, TPGA or TPGA@CZB hydrogel extract-supplemented medium in the lower chamber. Following 24-h incubation, migrated cells on the lower surface were fixed and stained with crystal violet. Cell migration was then observed under an inverted microscope.

### The role of TPGA@CZB in promoting cartilage formation *in vitro*

Adenocarcinoma-derived chondrogenic cell line 5 (ATDC5, purchased from SUNNCELL, Wuhan, China) were seeded in 12-well plates. After cell attachment, they were treated with 2 μM H_2_O_2_ for 2 h. The medium was then replaced with fresh medium containing extracts of TP, TPGA or TPGA@CZB hydrogel, with medium changes performed every two days for a total of 21 days. The relative expression level of the early chondrogenic differentiation marker SOX9 and the mid-to-late chondrogenic differentiation marker COL2A1 was detected by quantitative real-time PCR (qPCR) on Day 7 and Day 21, respectively. In addition, after 21 days of treatment, the cells were fixed and stained with Alcian Blue (for glycosaminoglycan, GAG, detection) and Safranin O (for proteoglycan visualization), respectively. Deposition of extracellular matrix components was subsequently analyzed under a fluorescence microscope.

### Construction of a rat model of acute rotator cuff tear and repair

Male Sprague Dawley (SD) rats were anesthetized and a 1–1.5 cm longitudinal incision was created along the lateral acromion to expose the supraspinatus tendon. Following sharp transection of the supraspinatus tendon, a bone groove was created at the greater tuberosity. In the experimental group, the bone groove was injected with 100 μl of hydrogel before the retracted tendon stump was repositioned over the defect and secured with 6-0 nonabsorbable sutures. Control rats (Suture) were injected with 100 μl saline instead of hydrogel. Meanwhile, normal rats were used as the normal control group (Normal). All animal experiments were approved by the Laboratory Animal Ethics Committee of the Shenzhen PKU-HKUST Medical Center (Approval No. 2023-975).

### *In vivo* assessment of TPGA@CZB in accelerating rotator cuff tendon regeneration

The experimental rats were divided into 4 groups: normal group (Normal), saline-treated model group (Suture), TPGA-treated model group (Suture + TPGA) and TPGA@CZB-treated model group (Suture + TPGA@CZB). The rats were executed at 8 weeks postoperatively, and shoulder tissues were collected for histological analysis by H&E staining, Masson staining, Safranin O-Fast Green staining, Toluidine Blue staining, Sirius red staining, as well as immunohistochemical analyses to assess the rotator cuff repair effect. In addition, immunofluorescence analysis of Runt-related transcription factor 2 (Runx-2), M1 macrophage marker (iNOS) and M2 macrophage marker (CD206) in tissue samples was performed to assess the role of TPGA@CZB in promoting osteogenic differentiation and modulating the inflammatory microenvironment.

### *In vivo* biodegradation behavior and toxicity assessment

To evaluate the *in vivo* biodegradation behavior and toxicity of TPGA@CZB, a 28-day subcutaneous implantation experiment was conducted in SD rats. Briefly, after anesthetizing the rats, cylindrical hydrogels with a diameter of 0.6 cm and height of 0.2 cm were implanted subcutaneously into the dorsal region. After 2 and 4 weeks of implantation, the rats were euthanized and the remaining hydrogels were retrieved to observe the extent of degradation. Simultaneously, skin tissues from the implantation site and major organs were collected, and H&E staining was performed to assess the *in vivo* toxicity of the hydrogel.

### Statistical analysis

Data are presented as means ± SD from three or more independent experiments. Where applicable, Student’s *t*-test or one-way ANOVA (SPSS) was used for statistical comparison, with asterisks indicating *P < 0.05, **P < 0.01 and ***P < 0.001.

## Results and discussions

### Fabrication and characterization of PEGDA, GelMA-CPBA and CZB

**Figure 2:**
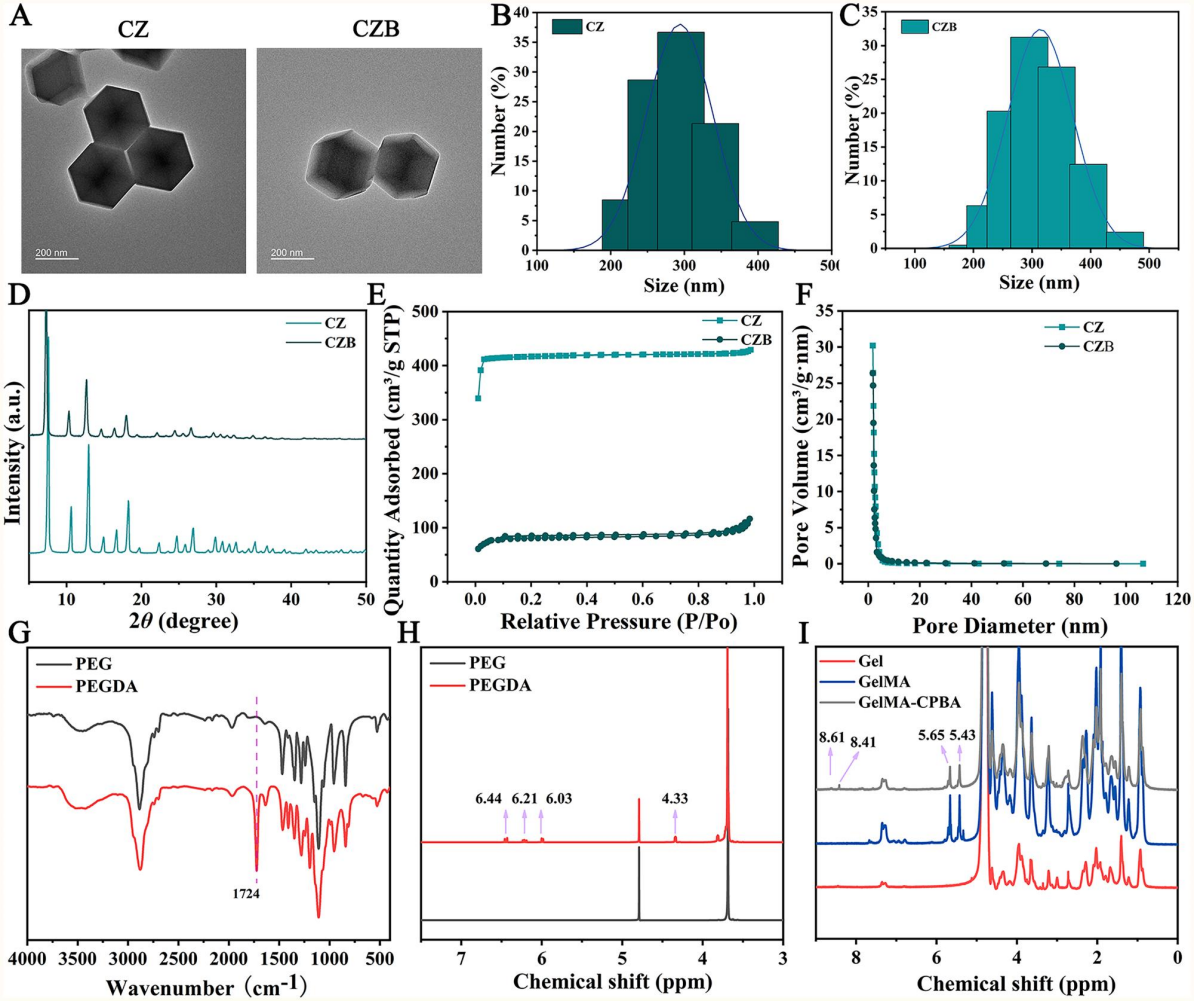
(**A**) Representative TEM images of CZ and CZB. particle size distributions of (**B**) CZ and (**C**) CZB. (**D**) XRD patterns, (**E**) nitrogen sorption/desorption isotherm and (**F**) pore size distributions for CZ as well as CZB. (**G**) FTIR and (**H**) ^1^H NMR spectra for PEGDA. (**I**) ^1^H NMR spectra for GelMA-CPBA.

The CZ was prepared by coprecipitation method, and then, Ba was loaded onto the CZ through electrostatic adsorption to obtain CZB. The loading ratio of Ba was detected to be 14.07 ± 2.11%. TEM revealed that the resulting CZ and CZB exhibited a dodecahedral structure similar to ZIF-8, with a particle size of approximately 300 nm (Figure 2A–C). XRD patterns showed that CZ displayed characteristic peaks at 2*θ *= 7.54°, 10.57°, 12.92° and 18.29°, consistent with the XRD patterns of ZIF-8 reported in the literature [12] (Figure 2D). The XRD pattern of CZB exhibited a slight leftward shift in the characteristic peaks, which may be attributed to the introduction of Ba causing minor lattice expansion. BET analysis indicated that after Ba loading, the pore volume of CZB significantly decreased compared to that of CZ (Figure 2E and F). The specific surface areas of CZ and CZB, as determined by the BET equation, were 1806.03 m^2^/g and 335.47 m^2^/g, respectively. These results demonstrate the successful preparation of CZB nanoparticles.

To prepare TPGA@CZB, PEGDA and GelMA-CPBA were synthesized. As depicted in Figure 2G, a strong absorption peak at 1724 cm^−1^ was observed in PEGDA’s FTIR spectrum, assigned to the ester C = O stretching vibration in the acrylate moiety, demonstrating successful DA grafting onto PEG. Meanwhile, in the ^1^H NMR spectrum of PEGDA, the characteristic peaks at 6.44 ppm, 6.21 ppm and 6.03 ppm were assigned to the olefinic protons in the acrylate group (CH_2_=CH−COO−), while the characteristic peak at 4.33 ppm was attributed to the methylene group (−O−CH_2_−) linked to the ester group [34] (Figure 2H). These results demonstrate the successful synthesis of PEGDA. The ^1^H NMR spectrum of GelMA-CPBA revealed characteristic peaks at 5.65 ppm and 5.43 ppm, confirming the successful introduction of double bonds onto Gel. Additionally, signals at 8.61 ppm and 8.41 ppm corresponded to aromatic protons from the phenylboronic acid moiety, verifying the successful incorporation of the boronic acid group [35, 36] (Figure 2I). These results demonstrate the successful synthesis of GelMA-CPBA.

### Fabrication and characterization of TPGA@CZB

**Figure 3:**
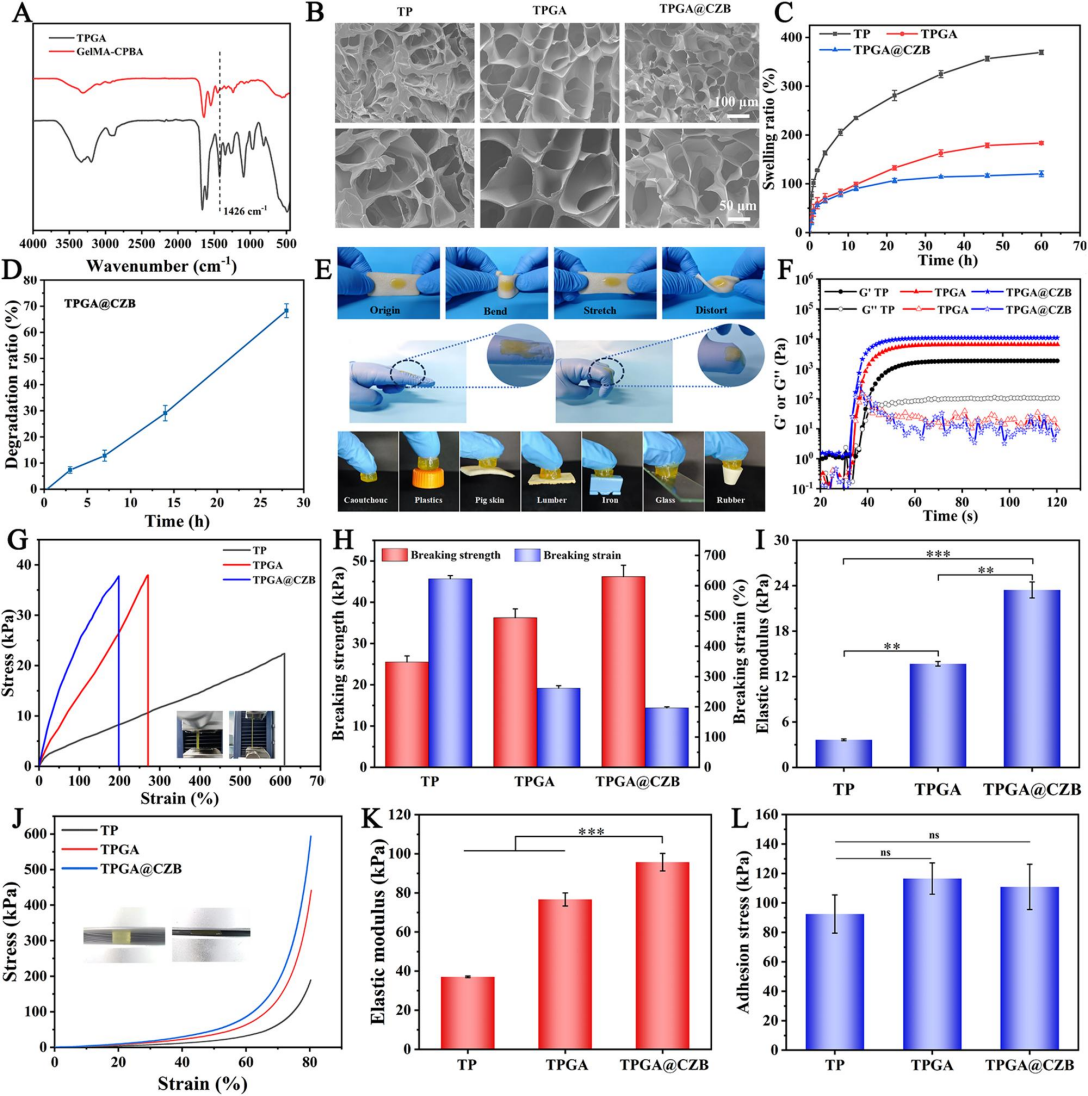
(**A**) FTIR spectra of TPGA and GelMA-CPBA. (**B**) Representative SEM images of TP, TPGA and TPGA@CZB. (**C**) Swelling curves of TP, TPGA and TPGA@CZB. (**D**) Degradation curve of TPGA@CZB. (**E**) Photographs of TPGA@CZB on pigskin, finger joints and adhesion on different substrates. (**F**) Rheological properties of TP, TPGA and TPGA@CZB. (**G**) The tensile stress–strain curves of TP, TPGA and TPGA@CZB, along with their corresponding (**H**) breaking strength, breaking strain and (**I**) elastic modulus. (**J**) The compressive stress–strain curves of TP, TPGA and TPGA@CZB, along with their corresponding (**K**) elastic modulus. (**L**) The adhesion strength of TP, TPGA and TPGA@CZB. *n *= 5. *p < 0.05, **p < 0.01, ***p < 0.001.

To provide a suitable environment for rotator cuff repair, CZB was encapsulated into a highly elastic and adhesive hydrogel formed by THMA, PEGDA and GelMA-CPBA, resulting in the preparation of TPGA@CZB. FTIR testing was conducted on the TPGA precursor solution to evaluate the formation of borate bonds. As shown in Figure 3A, a strong absorption peak at 1426 cm^−1^ appeared in the FTIR spectrum of the TPGA precursor solution, indicating the formation of B-O bonds. The microstructure of the hydrogel was observed via SEM, revealing that TPGA@CZB possesses a uniform porous network structure with a denser network compared to TP and TPGA (Figure 3B). Swelling tests demonstrated that the TP hydrogel exhibited high swelling behavior, with a swelling ratio of 369.46 ± 4.47%. In comparison, the swelling ratios of TPGA and TPGA@CZB decreased to 183.54 ± 3.29% and 120.45 ± 5.98%, respectively (Figure 3C). This decrease may be attributed to the increased hydrogel network density, which restricts water diffusion within the hydrogel. The lower swelling ratio is beneficial for extending the hydrogel’s service life and reducing compression on surrounding tissues, making TPGA@CZB suitable for rotator cuff repair applications. Additionally, the degradation tests revealed that TPGA@CZB underwent gradual degradation over 4 weeks, with a degradation ratio of 62.97 ± 0.94% on Day 28 (Figure 3D). These results demonstrate that TPGA@CZB can serve as a temporary scaffold to provide mechanical support during the initial stages of tendon–bone healing, while its subsequent gradual degradation prevents long-term foreign body reactions. To evaluate the adhesive capability and deformation resistance of TPGA@CZB, it was applied onto pigskin and finger joints followed by photo-curing. The results demonstrated that the hydrogel remained intact without detachment or fracture under various conditions, including bending, stretching and twisting of the pigskin, as well as during finger extension and flexion (Figure 3E). This indicates that the hydrogel possesses excellent adhesion and flexibility, enabling it to adapt to joint movements and maintain continuous contact at the tendon–bone interface. Rheological tests demonstrated that TPGA@CZB rapidly underwent gelation under UV irradiation. Compared to TP and TPGA, TPGA@CZB exhibited a higher storage modulus (*G*’), reaching approximately 10 kPa, indicating its superior resistance to deformation (Figure 3F). Furthermore, tensile and compression tests revealed the superior mechanical properties of TPGA@CZB. In the tensile test, TPGA@CZB exhibited excellent fracture resistance and stretchability, with a tensile fracture strength of 46.22 ± 2.74 kPa and a fracture strain of 196.24 ± 3.87% (Figure 3G–I). In the compression test, TPGA@CZB remained intact even at 80% strain, demonstrating its exceptional compressive resistance and toughness (Figure 3J). Additionally, the tensile elastic modulus and compressive elastic modulus of TPGA@CZB were 23.43 ± 1.06 kPa and 95.71 ± 4.47 kPa, respectively, indicating its favorable deformation resistance (Figure 3I and K). Furthermore, the adhesive capability of TPGA@CZB was quantified through lap shear testing. The results demonstrated that TPGA@CZB exhibits strong adhesive properties, achieving an adhesion strength of 110.90 ± 15.38 kPa to porcine skin (Figure 3L). This can be primarily attributed to the energy dissipation from the high-density hydrogen bonds in THMA and the enhanced interfacial interactions. This characteristic enables TPGA@CZB to function as a mechanical anchor at the tendon–bone interface and optimize stress distribution, thereby reinforcing tendon–bone integration. Collectively, these results confirm the successful fabrication of a viscoelastic hydrogel with outstanding adhesive and mechanical properties.

### Release properties of TPGA@CZB

**Figure 4:**
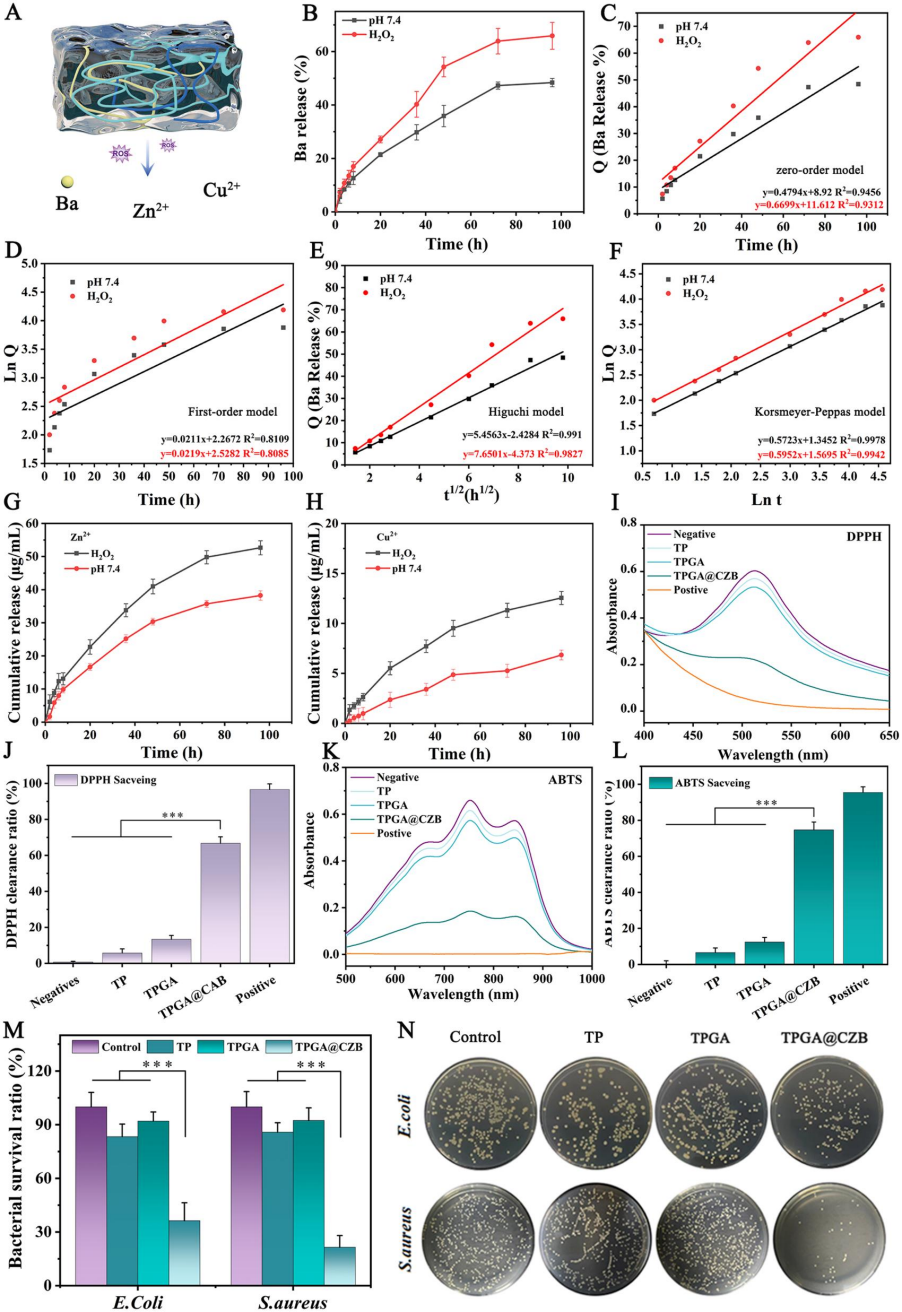
(**A**) Schematic diagram of ROS-responsive release of TPGA@CZB. (**B**) Release profiles of Ba in TPGA@CZB and their corresponding fittings using (**C**) zero-order, (**D**) first-order, (**E**) Higuchi and (**F**) Korsmeyer–Peppas models. Release curves of (**G**) Zn^2+^ and (**H**) Cu^2+^ in TPGA@CZB. (**I**) UV–Vis spectra along with (**J**) DPPH clearance ratio in the DPPH test. (**K**) UV–Vis spectra along with (**L**) ABTS clearance ratio for the ABTS test. (**M**) Bacterial survival and (**N**) photographs of colonies after TP, TPGA and TPGA@CZB treatments. *n *= 5.

The tendon–bone interface microenvironment is typically characterized by elevated ROS levels due to ischemic injury, inflammatory cell infiltration and mechanical loading. The ROS-responsive release behavior of TPGA@CZB was evaluated in PBS solutions containing H_2_O_2_ (Figure 4A). Results demonstrated that H_2_O_2_ significantly accelerated the release of Ba, Zn^2+^ and Cu^2+^ from TPGA@CZB (Figure 4B–H). In PBS alone, the cumulative release of Ba was 48.39 ± 1.56% at 96 h, whereas in H_2_O_2_-containing PBS, the release increased to 63.90 ± 4.76% at the same time point (Figure 4B). This acceleration primarily resulted from ROS-induced cleavage of borate ester bonds, which facilitated drug release. Furthermore, the release mechanism of TPGA@CZB was investigated through mathematical modeling. As shown in Figure 4C–F, the release profile of TPGA@CZB was optimally fitted to the Korsmeyer–Peppas model, suggesting that the release involves diffusion and solubilization control. These results demonstrated that TPGA@CZB possesses promising ROS-responsive release properties, enabling controlled drug delivery for tendon–bone interface repair.

### Antioxidant and antimicrobial properties of TPGA@CZB

Excessive ROS causes cellular and extracellular matrix damage, exacerbates chronic inflammation and delays tendon-to-bone healing. Therefore, the antioxidant capacity of hydrogels plays a critical role in maintaining microenvironmental stability and promoting tendon–bone healing. DPPH and ABTS assays demonstrated that TPGA@CZB was able to effectively scavenges free radicals, with a clearance ratio of 66.75 ± 3.58% for DPPH and 74.73 ± 4.31% for ABTS^+^**·** (Figure 4I–L). These results reveal the great potential of TPGA@CZB in promoting tendon-to-bone healing by mitigating oxidative stress.

Next, the antibacterial capability of TPGA@CZB was evaluated using *E. coli* and *S. aureus*. As depicted in Figure 4M and N, TPGA@CZB exhibits excellent antibacterial properties. After 8 h of co-culture with TPGA@CZB, the survival ratio of *E. coli* and *S. aureus* were significantly reduced to 36.35 ± 10.02% and 21.55 ± 6.48%, respectively. These findings indicate that TPGA@CZB possesses remarkable antimicrobial activity, which can effectively reduce infection risks associated with material implantation and help maintain local microenvironment stability in tendon-to-bone healing applications.

### *In vitro* biocompatibility of TPGA@CZB

**Figure 5:**
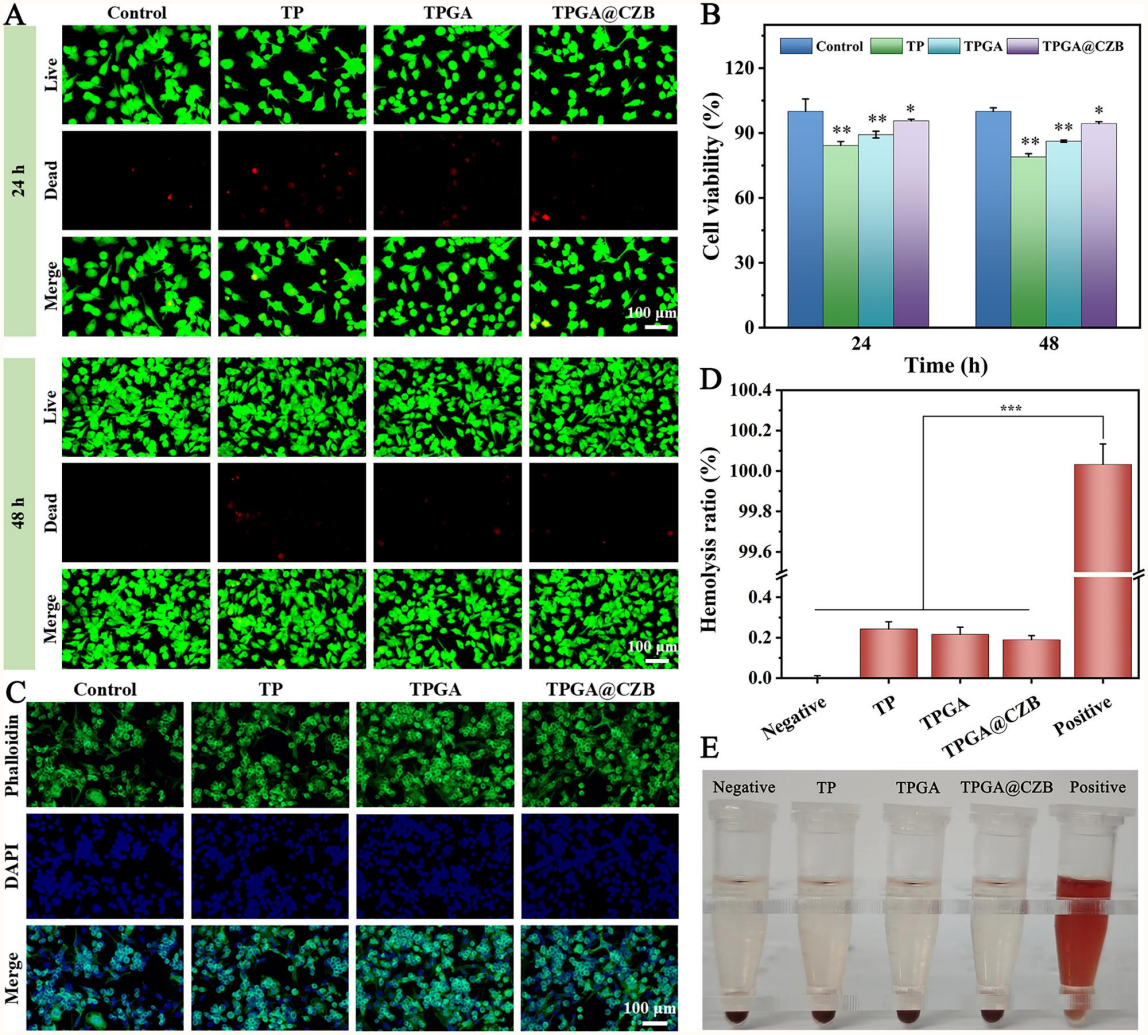
(**A**) Live/dead cell staining images, (**B**) cell activity detected by cck-8 assay and (**C**) cytoskeleton staining images of MC3T3-E1 cells co-cultured with TP, TPGA and TPGA@CZB. (**D**) Hemolysis ratio and (**E**) photographs of mouse erythrocytes co-cultured with TP, TPGA and TPGA@CZB. *n *= 5.

The biocompatibility of TPGA@CZB was evaluated to validate its applicability in tissue engineering. Live/dead cell staining revealed that MC3T3-E1 cells co-cultured with TPGA@CZB maintained high survival ratio at both 24 and 48 h, with no significant difference in cell death compared to the control group (Figure 5A). Correspondingly, the cell viability of MC3T3-E1 cells cultured with TPGA@CZB remained above 90% at both 24 and 48 h, confirming negligible cytotoxic effects (Figure 5B). Besides, cytoskeletal staining revealed that MC3T3-E1 cells co-cultured with TPGA@CZB displayed well-spread morphology and increased cell density, reflecting positive impacts on cellular adhesion and growth (Figure 5C). Furthermore, hemolysis analysis revealed that TPGA@CZB caused no significant hemolysis in mouse erythrocytes during co-culture, with a hemolysis ratio of merely 0.2% (Figure 5D and E). Collectively, these results confirm the excellent biocompatibility of TPGA@CZB, supporting its potential for *in vivo* applications in promoting tendon-to-bone healing.

### Intracellular ROS scavenging and *in vitro* modulation of macrophage polarization by TPGA@CZB

**Figure 6:**
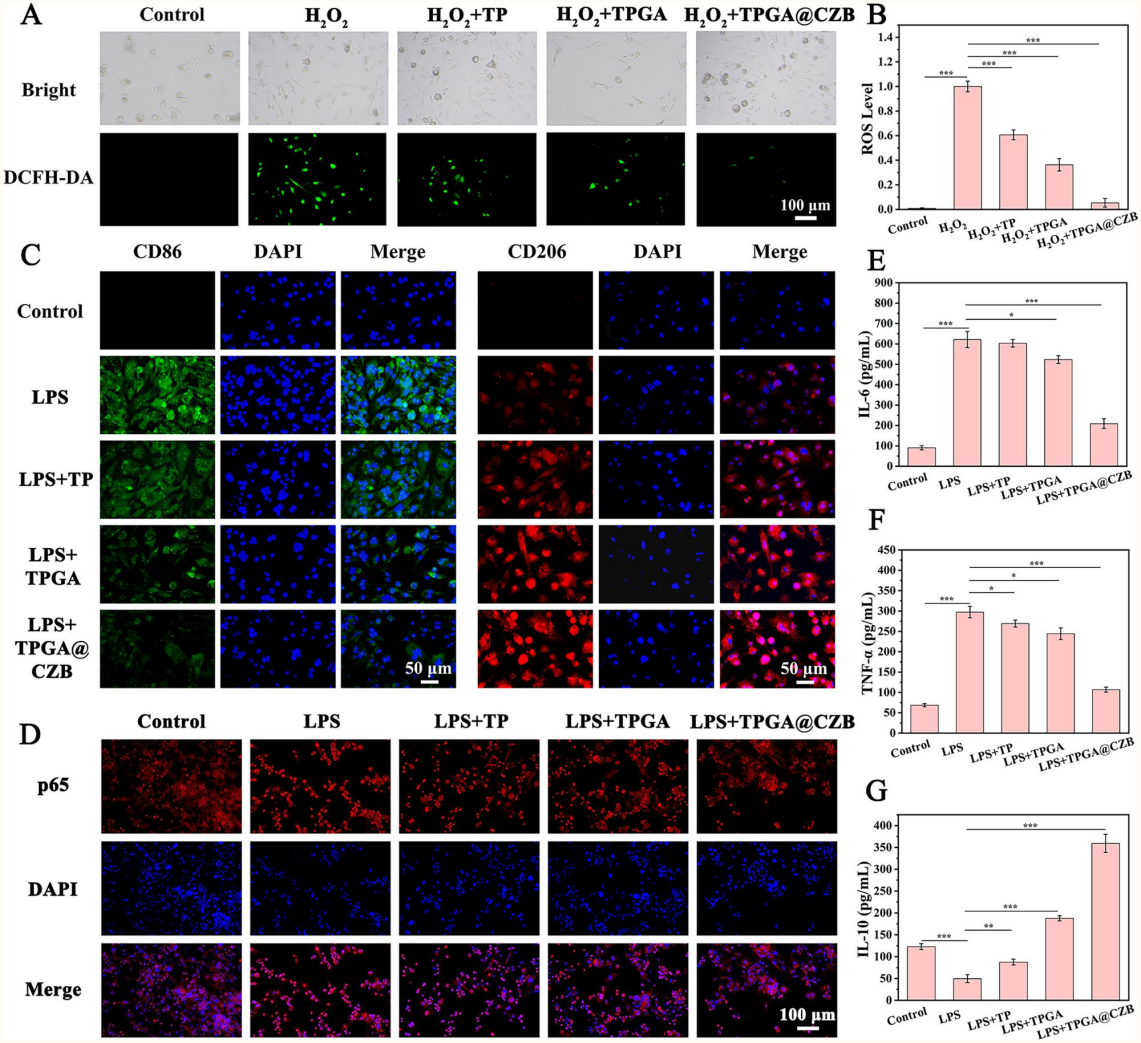
(**A**) Intracellular ROS staining images of MC3T3-E1 cells. (**B**) Quantification of intracellular ROS levels. (**C**) Fluorescence microscopy images of CD86 and CD206 in RAW264.7 cells under different treatment conditions. (**D**) Immunofluorescence staining image of p65. Expression levels of (**E**) IL-6, (**F**) TNF-α and (**G**) IL-10 in macrophage conditioned medium. *n *= 5.

The intracellular ROS scavenging ability of TPGA@CZB was investigated in H_2_O_2_-stimulated MC3T3-E1 cells. As shown in Figure 6A and B, H_2_O_2_-treated MC3T3-E1 cells exhibited substantial ROS generation. In contrast, TPGA@CZB treatment significantly reduced intracellular ROS levels in H_2_O_2_-exposed MC3T3-E1 cells. These results demonstrate that TPGA@CZB possesses potent intracellular ROS scavenging capacity, suggesting its potential to effectively promote tendon-to-bone healing by mitigating cellular oxidative stress.

Macrophages are key regulators of the inflammatory microenvironment and their polarization state is critical during tissue repair. Herein, the regulatory role of TPGA@CZB in macrophage polarization was investigated using LPS-activated RAW264.7 cells. As shown in Figure 6C, LPS treatment significantly promote the polarization of RAW264.7 cells towards the pro-inflammatory M1 phenotype, demonstrated by a pronounced increase in CD86 expression. In contrast, TPGA@CZB treatment significantly suppressed CD86 expression while enhancing CD206 expression in LPS-activated RAW264.7 cells. These results demonstrate that TPGA@CZB can reprogram macrophages from the M1 (pro-inflammatory) to the M2 (anti-inflammatory) phenotype, highlighting its role in controlling inflammation and promoting tissue regeneration in tendon–bone healing. Given the central role of the NF-κB signaling pathway in regulating macrophage polarization, the modulatory effects of TPGA@CZB on this pathway were investigated. Immunofluorescence staining for p65 (a core NF-κB component) revealed a significant increase in nuclear translocation of p65 in LPS-induced RAW264.7 cells, indicating NF-κB pathway activation. TPGA@CZB treatment effectively reduced LPS-induced p65 nuclear translocation, demonstrating its ability to inhibit NF-κB pathway activation (Figure 6D). Furthermore, ELISA analysis of conditioned medium from RAW264.7 cells revealed that TPGA@CZB effectively suppressed the expression of proinflammatory factors (IL-6 and TNF-α) while promoting the expression of the anti-inflammatory factor (IL-10) in LPS-induced cells (Figure 6E–G). These findings indicate that the regulatory effect of TPGA@CZB on macrophage polarization is associated with the NF-κB signaling pathway. By inhibiting NF-κB signaling and reprogramming macrophage polarization, TPGA@CZB facilitates the establishment of an anti-inflammatory microenvironment conducive to tendon–bone healing.

### TPGA@CZB promotes angiogenesis *in vitro*

**Figure 7:**
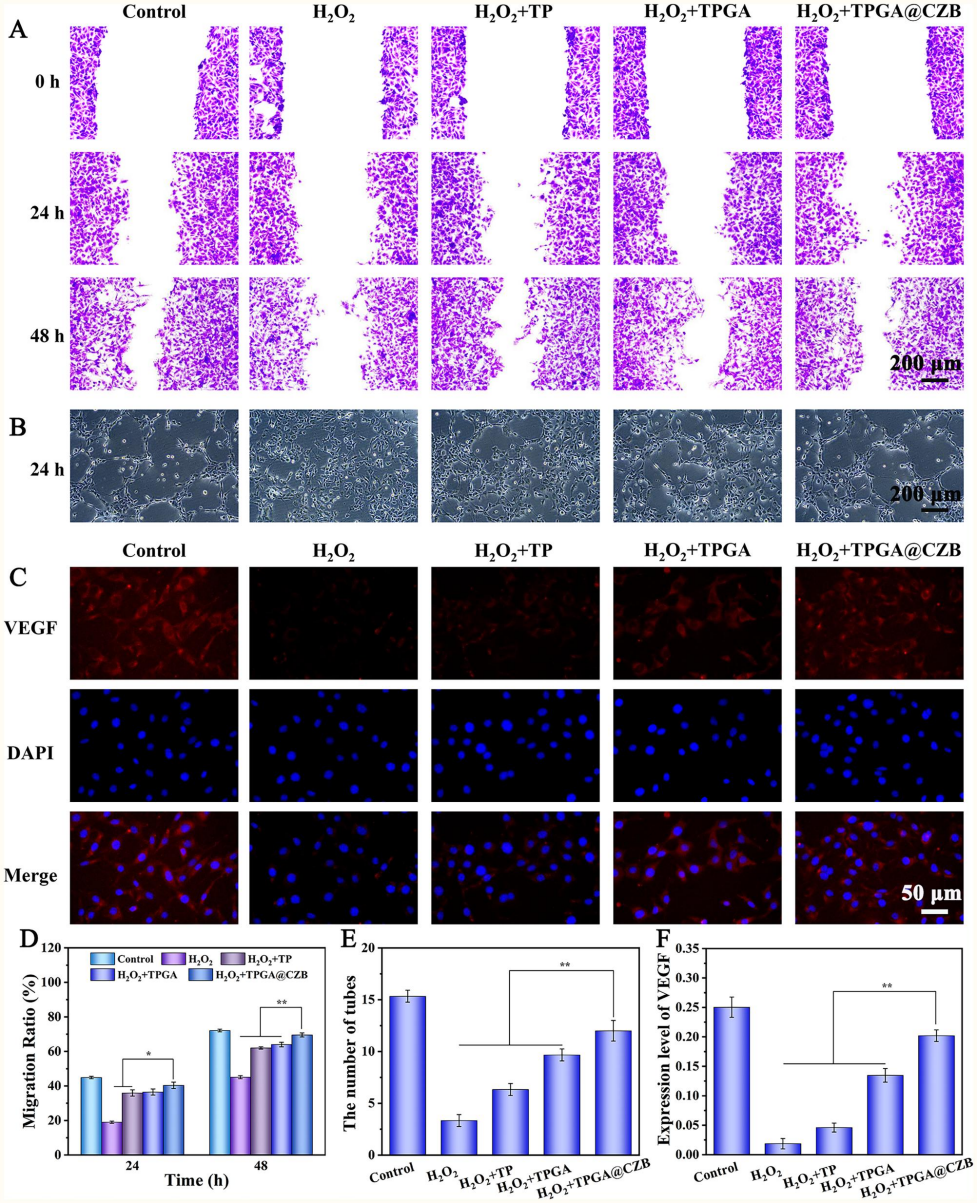
(**A**) Scratch assay images, (**B**) tube formation assay images and (**C**) VEGF immunofluorescence images of HUVECs. (**D**) Migration ratio of HUVECs from different treatment groups in the scratch assay. (**E**) Number of tubes formed by HUVECs from different treatment groups in the tube formation assay. (**F**) VEGF expression levels in HUVECs subjected to different treatments. *n *= 5.

The pro-angiogenic effects of TPGA@CZB in tendon–bone healing were investigated using H_2_O_2_-stimulated HUVECs. Scratch assays and tube formation assays demonstrated that H_2_O_2_ stimulation significantly impaired the migration and tube-forming capabilities of HUVECs, whereas TPGA@CZB treatment largely restored these functions in H_2_O_2_-stimulated HUVECs (Figure 7A, B, D and E). Additionally, immunofluorescence staining revealed that VEGF, a key regulator of angiogenesis, was significantly upregulated in the TPGA@CZB co-treatment group compared to the H_2_O_2_ group (Figure 7C and F). These findings demonstrate that TPGA@CZB can effectively promote angiogenesis, which may be attributed to the remarkable pro-angiogenic properties of Cu^2+^ and the synergistic effects of Ba and Zn^2+^. By promoting proper vascularization during the initial healing phase of the tendon-to-bone bond, TPGA@CZB is able to deliver oxygen and nutrients to the repair site and helps to modulate the inflammatory environment. In the later healing stage, as oxidative stress levels decrease at the injury site, reduced drug release prevents excessive angiogenesis, thereby avoiding scar tissue formation.

### TPGA@CZB promotes osteogenic differentiation and chondrogenesis *in vitro*

**Figure 8:**
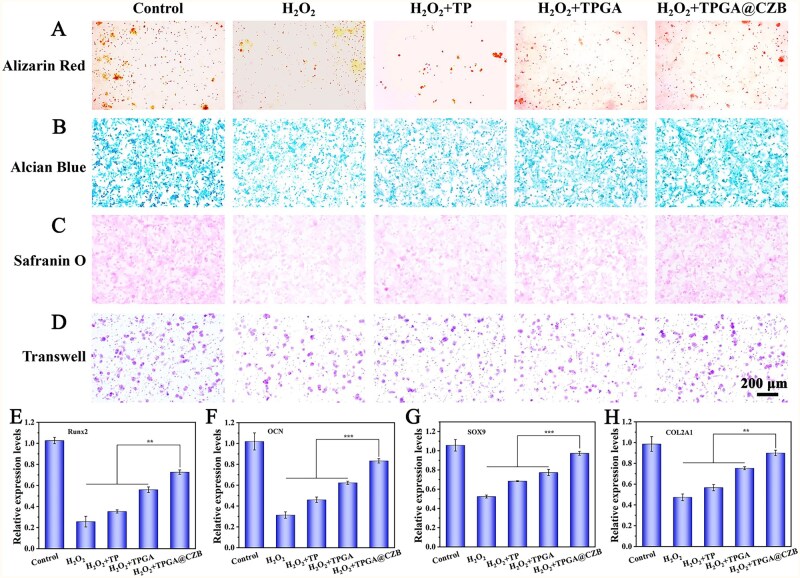
(**A**) ARS staining images of MC3T3-E1 cells. (**B**) Alisin blue staining images and (**C**) saffron O staining images of ATDC5 cells. (**D**) Transwell images of MC3T3-E1 cells. Relative expression levels of (**E**) Runx2 and (**F**) OCN in MC3T3-E1 cells. Relative expression levels of (**G**) SOX9 and (**H**) COL2A1 in ATDC5 cells. *n *= 5.

The effective healing of the tendon–bone interface depends on the regeneration and bony integration of the fibrocartilage band, a process that requires the synergistic effect of cartilage formation and osteogenic differentiation. In this study, H_2_O_2_-stimulated MC3T3-E1 cells were used to investigate the effect of TPGA@CZB on osteogenic differentiation under oxidative stress conditions. ARS staining revealed that H_2_O_2_-stimulated MC3T3-E1 cells exhibited minimal calcium deposition, whereas those co-treated with TPGA@CZB and H_2_O_2_ showed a greater number and larger size of mineralized nodules, with calcium deposition levels comparable to the Control group (Figure 8A). Furthermore, qPCR results demonstrated that TPGA@CZB significantly upregulated the expression of Runx2 and OCN under oxidative stress (Figure 8E and F). These findings confirm that TPGA@CZB can mitigate oxidative stress at the tendon–bone injury site and promote osteogenic differentiation.

To evaluate the role of TPGA@CZB in promoting chondrogenesis, GAGs and Proteoglycans were detected in H_2_O_2_-stimulated ATDC5 cells. In alcian blue staining, ATDC5 cells co-treated with TPGA@CZB and H_2_O_2_ exhibited significantly more intense blue precipitation compared to the H_2_O_2_ group, indicating GAGs enrichment (Figure 8B). Meanwhile, in the safranin O staining, the TPGA@CZB and H_2_O_2_ co-treatment group showed markedly stronger red-positive staining relative to the H_2_O_2_ group, demonstrating enhanced proteoglycans synthesis (Figure 8C). Furthermore, qPCR results demonstrated that TPGA@CZB significantly upregulates the expression of SOX9 and COL2A1 in ATDC5 cells under oxidative stress (Figure 8G and H). These results demonstrate the positive role of TPGA@CZB in promoting cartilage differentiation.

Moreover, insufficient cell migration is a critical limiting factor in tendon–bone interface repair. Transwell migration assay revealed that TPGA@CZB significantly enhanced the migratory ability of MC3T3-E1 cells under oxidative stress conditions. In the group treated with TPGA@CZB and H_2_O_2_, the number of migrated cells increased by approximately 60% compared to the group treated with H_2_O_2_ alone (Figure 8D). Collectively, these findings reveal the potential of TPGA@CZB to enhance tendon–bone interface repair through facilitating cell migration, osteogenic differentiation and cartilage formation.

### TPGA@CZB promotes rotator cuff repair *in vivo*

**Figure 9:**
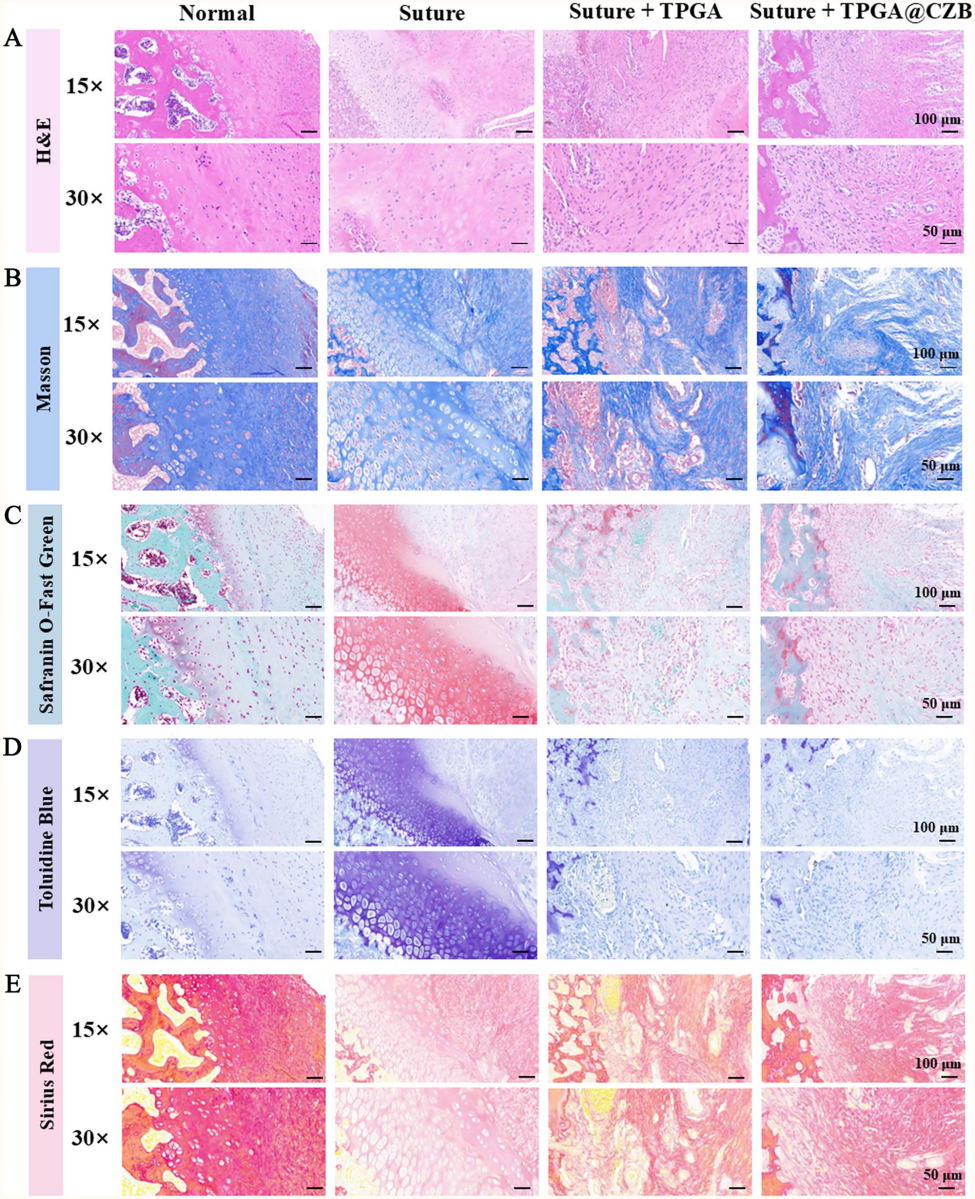
(**A**) H&E-stained images, (**B**) Masson-stained images, (**C**) Safranin O-Fast green-stained images, (**D**) Toluidine blue-stained images and (**E**) Sirius red-stained images of shoulder tissue sections from rats with different treatments. *n *= 6.

**Figure 10:**
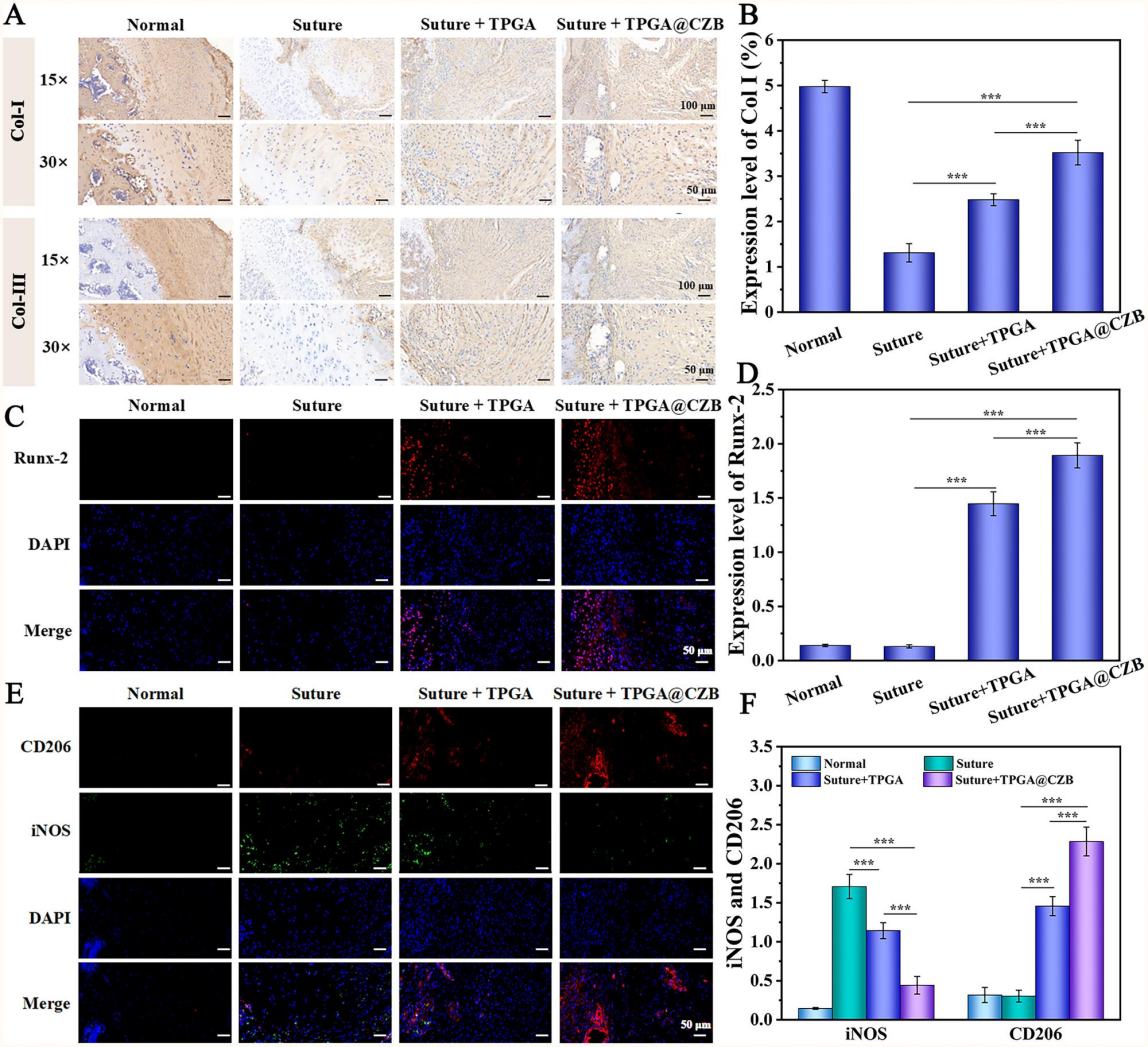
(**A**) Col-I and Col-III immunohistochemistry images. (**B**) Quantification of Col-I expression levels. (**C**) Runx-2 immunofluorescence images and (**D**) quantitative analysis. (**E**) Dual immunofluorescence staining for iNOS and CD206, along with (**F**) their quantitative analysis. *n *= 6.

To assess the effectiveness of TPGA@CZB in enhancing tendon–bone healing, shoulder tissues were harvested from euthanized rats 8 weeks after surgery for histological examination. H&E staining as well as Masson staining revealed that in the rat model of acute rotator cuff tear and repair, the TPGA@CZB group exhibited improved collagen tissue organization, featuring more densely packed and uniformly aligned collagen fibers at the interface (Figure 9A and B). In addition, Safranin O-Fast Green and Toluidine Blue staining showed fibrocartilaginous transition zone formation at the tendon–bone interface, which closely resembles the morphology of native tissue. (Figure 9C and D). During tendon-to-bone healing, the maturation of Col-I and subsequent degradation of Col-III are critical for restoring mechanical properties. Sirius red staining showed that the TPGA@CZB group exhibited more intense red coloration and a continuous gradient transitioning from light to dark red from the tendon to the bone, in contrast to both the control and TPGA groups, indicating an increase in Col-I content and higher maturity of collagen fibers (Figure 9E). Correspondingly, immunohistochemical analysis revealed elevated Col-I levels in the TPGA@CZB treatment group relative to both the control and TPGA groups, while Col-III levels showed a downward trend (Figure 10A and B). These results suggest that TPGA@CZB is able to optimize collagen remodeling and promote functional regeneration at the tendon–bone interface.

Runx-2 is a master transcription factor for osteogenic differentiation [37]. Immunofluorescence staining demonstrated that Runx-2 expression was significantly upregulated on the bone side in the TPGA@CZB treatment group, indicating the initiation of osteogenic differentiation in mesenchymal stem cells and tendon-derived cells (Figure 10C and D). Furthermore, macrophage polarization status in the tendon–bone interface was evaluated through immunofluorescence co-staining of CD206 and iNOS. The results showed that in rat models of acute rotator cuff tear and repair, TPGA@CZB treatment significantly increased CD206 expression while suppressed iNOS expression at the tendon–bone interface, demonstrating its ability to modulate macrophage polarization and ameliorate the inflammatory microenvironment (Figure 10E and F).

Collectively, the above results suggest that TPGA@CZB can effectively promote rotator cuff healing. This may be attributed to the multiple positive effects exerted by TPGA@CZB at the tendon–bone interface. On one hand, the viscoelastic hydrogel matrix with a porous structure provides essential mechanical support for tissue repair while serving as a scaffold for cell infiltration. On the other hand, TPGA@CZB releases active substances on demand in response to ROS levels. The released Ba, Zn^2+^ and Cu^2+^ perform various biological functions at the tendine-bone interface, including antioxidation, immune regulation, promoting angiogenesis and enhancing cell osteogenesis and cartilage differentiation. These synergistic effects effectively facilitate the formation of fibrocartilage matrix between tendons and bones, ultimately improving tendon-to-bone integration.

### *In vivo* biosafety and degradation properties of hydrogels

**Figure 11:**
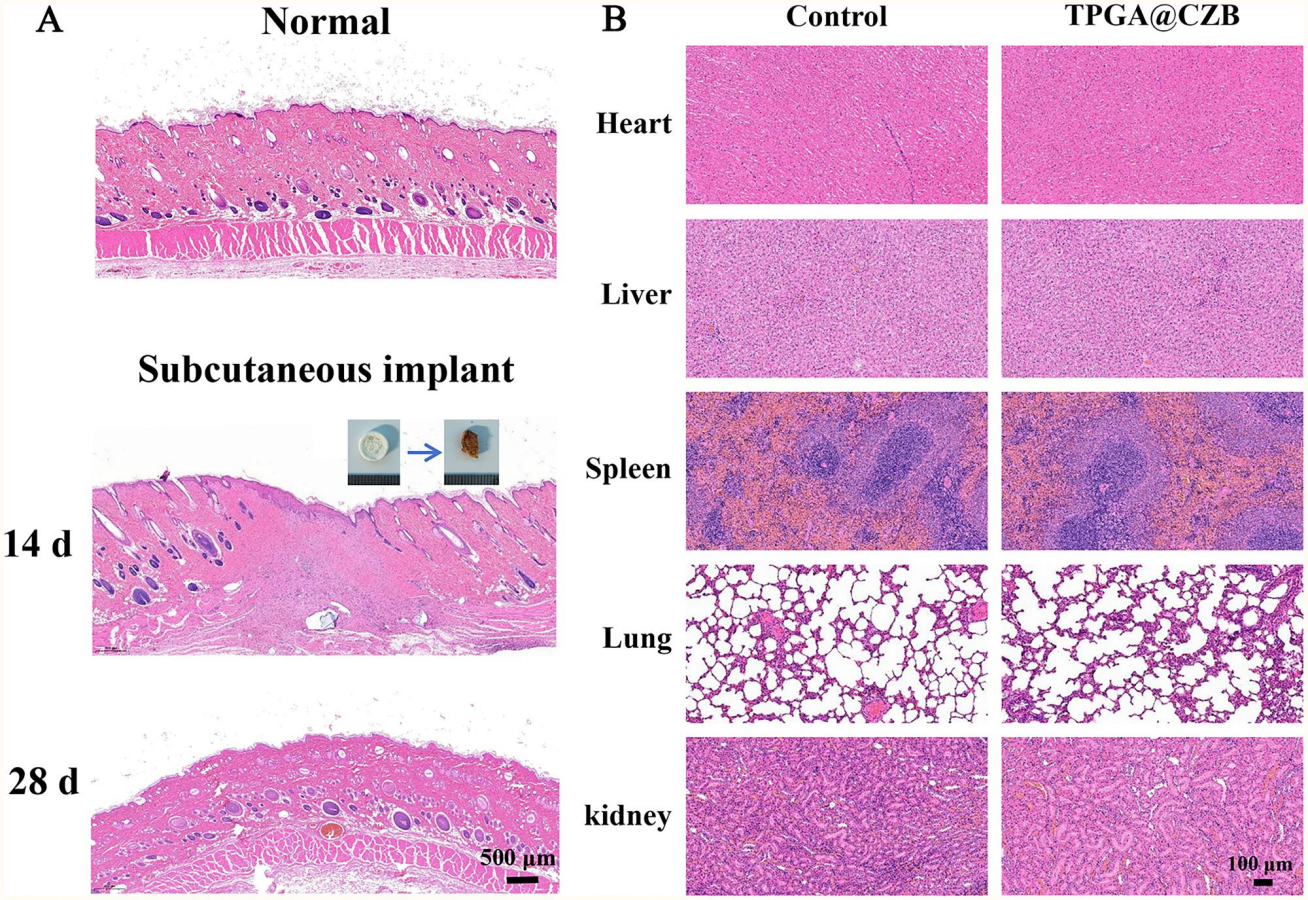
(**A**) H&E-stained images of normal skin tissue and skin tissue implanted with TPGA@CZB for 14 days and 28 days. (**B**) H&E-stained images of major organs in rats implanted with TPGA@CZB for 28 days. *n *= 6.

To evaluate the *in vivo* biodegradation behavior and toxicity of TPGA@CZB, a 28-day implantation experiment was conducted in rats. The results demonstrated that TPGA@CZB gradually degraded over the 28-day period alongside tissue repair, achieving tight integration with the surrounding tissues and forming a biocompatible interface (Figure 11A). Furthermore, major organs were collected after 28 days of subcutaneous implantation for H&E staining. The histological analysis revealed no pathological changes in the organs of rats treated with TPGA@CZB, indicating the absence of systemic toxicity (Figure 11B). These findings collectively suggest that TPGA@CZB possesses suitable degradation characteristics and excellent biocompatibility for tendon–bone healing applications.

## Conclusion

In conclusion, a novel ROS-responsive adhesive nanocomposite hydrogel (TPGA@CZB) was developed in this study for promoting regeneration at the tendon–bone interface. This system significantly enhanced the solubility and bioavailability of Ba by utilizing Cu-Zn MOF for its delivery, while synergistically leveraging the biological activities of Zn^2+^ and Cu^2+^ to exert multiple therapeutic functions. The hydrogel, constructed from THMA, PEGDA and GelMA-CPBA to form a multi-crosslinked network, exhibited excellent adhesive properties (lap shear strength of 110.90 ± 15.38 kPa) and mechanical adaptability (compressive strain > 80%), providing dynamic mechanical support to the injury site. Owing to the ROS-sensitive borate ester bonds, TPGA@CZB achieved precise controlled drug release in H_2_O_2_-rich environments, enhancing therapeutic efficiency and biosafety. *In vitro* and *in vivo* experiments confirmed that TPGA@CZB effectively modulates the inflammatory microenvironment, enhances osteogenic differentiation and chondrogenesis, as well as optimizes collagen remodeling, thereby facilitating functional regeneration of the tendon–bone interface. Thus, this multifunctional hydrogel—integrating “mechanical support, on-demand drug release and microenvironment regulation”—offers an innovative strategy with high clinical translation potential for tendon–bone interface regeneration.
